# 3-Hydr­oxy-3a,6,8c-trimethyl­perhydro­oxireno[2′,3′:7,8]naphtho[1,2-*b*]furan-7(2*H*)-one

**DOI:** 10.1107/S1600536809025124

**Published:** 2009-07-04

**Authors:** Victor Kesternich, Paulina Cortés, Iván Brito, Alejandro Cárdenas, Matías López-Rodríguez

**Affiliations:** a Departamento de Química, Universidad Católica del Norte, Casilla 1280, Antofagasta, Chile; b Departamento de Química, Facultad de Ciencias Básicas, Universidad de Antofagasta, Casilla 170, Antofagasta, Chile; c Departamento de Física, Facultad de Ciencias Básicas, Universidad de Antofagasta, Casilla 170, Antofagasta, Chile; d Instituto de Bio-Orgánica ’Antonio González’, Universidad de La Laguna, Astrofísico Francisco Sánchez N°2, La Laguna, Tenerife, Spain

## Abstract

The title compound, C_15_H_22_O_4_, consists of two *trans*-fused six-membered rings and a *trans*-fused five-membered γ-lactone. The ep­oxy and hydroxyl groups are α-oriented. The cyclo­hexane rings adopt half-chair and chair conformations and the lactone ring is in an envelope conformation. The mol­ecular structure is stabilized by one O—H⋯O and three C—H⋯O intra­molecular hydrogen bonds.

## Related literature

For background to sesquiterpene lactones, see: Fraga (2008 ▶). For their biological activity, see: Pillay *et al.* (2007 ▶); Ohno *et al.* (2005 ▶); Lindenmeyer *et al.* (2006 ▶). For synthetic details, see: Villar *et al.* (1983 ▶); González, *et al.* (1982 ▶). For a related structure, see: Rychlewska *et al.* (1982 ▶). For puckering parameters, see: Cremer & Pople (1975 ▶).
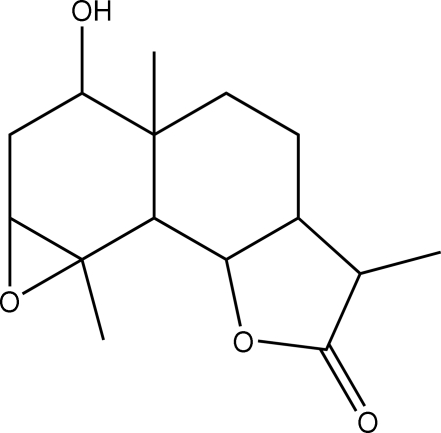


## Experimental

### 

#### Crystal data

C_15_H_22_O_4_
*M* *_r_* = 266.33
Monoclinic, 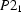

*a* = 8.251 (3) Å
*b* = 7.239 (2) Å
*c* = 11.434 (2) Å
β = 94.201 (5)°
*V* = 681.1 (3) Å^3^
*Z* = 2
Mo *K*α radiation
μ = 0.09 mm^−1^
*T* = 292 K
0.20 × 0.09 × 0.08 mm

#### Data collection

Nonius KappaCCD area-detector diffractometer
Absorption correction: none
6696 measured reflections
1601 independent reflections
1495 reflections with *I* > 2σ(*I*)
*R*_int_ = 0.042

#### Refinement

*R*[*F* ^2^ > 2σ(*F* ^2^)] = 0.037
*wR*(*F* ^2^) = 0.108
*S* = 1.13
1601 reflections
179 parameters
1 restraint
H atoms treated by a mixture of independent and constrained refinement
Δρ_max_ = 0.17 e Å^−3^
Δρ_min_ = −0.15 e Å^−3^

### 

Data collection: *COLLECT* (Nonius, 2000 ▶); cell refinement: *DENZO-SMN* (Otwinowski & Minor, 1997 ▶); data reduction: *DENZO-SMN*; program(s) used to solve structure: *SIR97* (Altomare *et al.*, 1999 ▶); program(s) used to refine structure: *SHELXL97* (Sheldrick, 2008 ▶); molecular graphics: *ORTEP-3 for Windows* (Farrugia, 1997 ▶) and *PLATON* (Spek, 2009 ▶); software used to prepare material for publication: *WinGX* (Farrugia, 1999 ▶).

## Supplementary Material

Crystal structure: contains datablocks global, I. DOI: 10.1107/S1600536809025124/pv2169sup1.cif

Structure factors: contains datablocks I. DOI: 10.1107/S1600536809025124/pv2169Isup2.hkl

Additional supplementary materials:  crystallographic information; 3D view; checkCIF report

## Figures and Tables

**Table 1:** Hydrogen-bond geometry (Å, °)

*D*—H⋯*A*	*D*—H	H⋯*A*	*D*⋯*A*	*D*—H⋯*A*
O4—H4⋯O3	0.79 (4)	2.27 (4)	2.952 (3)	146 (5)
C9—H9*A*⋯O4	0.97	2.55	2.958 (3)	105
C5—H5⋯O4	0.98	2.57	2.925 (3)	101
C15—H15*C*⋯O2	0.96	2.53	2.913 (3)	104

**Fig. 1.**
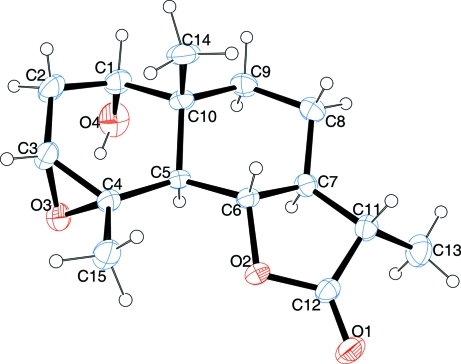
The molecular structure of (I). Displacement ellipsoids are drawn at the 30% probability level.

**Fig. 2.**
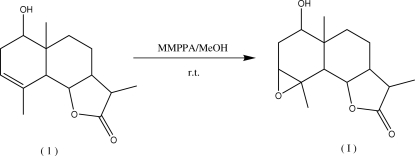
Preparation of the title compound.

## supplementary crystallographic information

### Comment

Sesquiterpene lactones constitute a large group of natural products (Fraga, 2008). The eudesmanolides are natural products belong to the sesquiterpene lactones composed of fifteen carbon atoms. Many of these compounds, natural or synthetics, are of particular interest because of their biological activity (Pillay, *et al.*, 2007; Ohno, *et al.*, 2005; Lindenmeyer, *et al.*, 2006). We report in this article the synthesis and crystal structure of a novel eudesmanolide, the title compound, (I).

The structure of the title compound (Fig. 1) is stabilized by one O—H···O and three C—H···O intramolecular hydrogen bonds (Table 1). The structure of (I) consists of two *trans*-fused [C(5)—C(10)] six-membered rings and a *trans*-fused [at C(6)—C(7)] five-membered γ-lactone. The epoxy and hidroxyl group are α -oriented. The cyclohexane rings adopt half-chair [C1/C2/C3/C4/C5/C10] and chair [C5/C6/C7/C8/C9/C10] and the lactone ring is in an envelope conformation respectively, as shown by the Cremer & Pople (1975) puckering parameters [Q_T_=0.525 (2) Å, θ =46.9 (2)°, φ=321.1 (4)°; Q_T_=0.616 (2) Å, θ =8.81 (2)°, φ=56.3 (1)°; q_2_=0.382 (2) Å, φ_2_=244.5 (3)°, respectively].

The crystal structure of the title compound is isomorphous with erivanin (Rychlewska *et al.*, 1982).

### Experimental

The title compound (I) was prepared by epoxidation of 1 with monoperoxyphthalic acid magnesium (MMPPA) at room temperature as shown in Fig. 2. In turn the product 1 was obtained by reduction of desoxyvulgarina(1-oxo-6β,7α,11β-H-eudesm-4-en-6,12-olide) (Villar *et al.*, 1983), with sodium borohydride in ethanol (González, *et al.*, 1982). Recrystallization from hexane/athyl acetate (3:1) at room temperature afforded colourless crystals suitable for X-ray diffraction analysis.

### Refinement

Due to lack of sufficient anomalous dospersion effects, an absolute structure was not established. Therefore, Friedel pairs (634) were merged. The hydroxyl H4 atom was located in Fourier difference maps and refined isotropically. All other H atoms were positioned geometrically and treated as riding with C—H = 0.98 Å for CH, 0.97 Å (CH_2_) or 0.96 Å (CH_3_) with *U*_iso_(H) = 1.2 *U*_eq_ (C) (for CH, CH_2_) or *U*_iso_(H) = 1.5*U*_eq_(C) (for CH_3_).

### Crystal data

**Table:**

C_15_H_22_O_4_	*F*(000) = 288
*M**_r_* = 266.33	*D*_x_ = 1.299 Mg m^−^^3^
Monoclinic, *P*2_1_	Mo *K*α radiation, λ = 0.71069 Å
Hall symbol: P 2yb	Cell parameters from 1601 reflections
*a* = 8.251 (3) Å	θ = 2.4–27.1°
*b* = 7.239 (2) Å	µ = 0.09 mm^−^^1^
*c* = 11.434 (2) Å	*T* = 292 K
β = 94.201 (5)°	Block, colourless
*V* = 681.1 (3) Å^3^	0.20 × 0.09 × 0.08 mm
*Z* = 2	

### Data collection

**Table:**

Nonius KappaCCD area-detector diffractometer	1495 reflections with *I* > 2σ(*I*)
Radiation source: fine-focus sealed tube	*R*_int_ = 0.042
graphite	θ_max_ = 27.1°, θ_min_ = 2.5°
φ scans, and ω scans with κ offsets	*h* = −8→10
6696 measured reflections	*k* = −9→9
1601 independent reflections	*l* = −14→14

### Refinement

**Table:**

Refinement on *F*^2^	Primary atom site location: structure-invariant direct methods
Least-squares matrix: full	Secondary atom site location: difference Fourier map
*R*[*F*^2^ > 2σ(*F*^2^)] = 0.037	Hydrogen site location: inferred from neighbouring sites
*wR*(*F*^2^) = 0.108	H atoms treated by a mixture of independent and constrained refinement
*S* = 1.13	*w* = 1/[σ^2^(*F*_o_^2^) + (0.0654*P*)^2^ + 0.0443*P*] where *P* = (*F*_o_^2^ + 2*F*_c_^2^)/3
1601 reflections	(Δ/σ)_max_ < 0.001
179 parameters	Δρ_max_ = 0.17 e Å^−^^3^
1 restraint	Δρ_min_ = −0.15 e Å^−^^3^

### Special details

**Table:**

Experimental. Melting points were determined on a Kofler-type apparatus and are uncorrected. The IR spectra were recorded on a Perkin-Elmer Spectrum BX spectrophotometer with KBr as support. The ^1^H-NMR spectra were obtained with a Brüker Advance DPX-400 at 400 MHz. The MS spectra were recorded on a VG AUTOSPEC FISON instrument. In the purification of the intermediates and final product column chromatography was carried out using Merck silica gel 0.065–0.2 mm. Melting point 473–475 K. IR cm^-1^: 3529 (O—H), 1769 (γ-lactone). ^1^H-NMR, δ (CDCl_3_): 3.96 (1*H*, dd, J=9.7 and 8.5 Hz, H6), 3.21 (1*H*, bs, H1), 3.03 (1*H*, bs, H3), 1.49 (3*H*, s, H15), 1.24 (3*H*, d, J= 6.8 Hz, H13), 1.21 (3*H*, s, H14). MS (m/*z*): 266.15 (*M*^+^, C_15_H_22_O_4_), 248.14 (*M*^+^– H_2_O).
Geometry. All s.u.'s (except the s.u. in the dihedral angle between two l.s. planes) are estimated using the full covariance matrix. The cell s.u.'s are taken into account individually in the estimation of s.u.'s in distances, angles and torsion angles; correlations between s.u.'s in cell parameters are only used when they are defined by crystal symmetry. An approximate (isotropic) treatment of cell s.u.'s is used for estimating s.u.'s involving l.s. planes.
Refinement. Refinement of *F*^2^ against ALL reflections. The weighted *R*-factor *wR* and goodness of fit *S* are based on *F*^2^, conventional *R*-factors *R* are based on *F*, with *F* set to zero for negative *F*^2^. The threshold expression of *F*^2^ > 2σ(*F*^2^) is used only for calculating *R*-factors(gt) *etc*. and is not relevant to the choice of reflections for refinement. *R*-factors based on *F*^2^ are statistically about twice as large as those based on *F*, and *R*- factors based on ALL data will be even larger.

### Fractional atomic coordinates and isotropic or equivalent isotropic displacement parameters (Å^2^)

**Table:**

	*x*	*y*	*z*	*U*_iso_*/*U*_eq_	
O1	0.6128 (2)	0.3084 (3)	0.41480 (13)	0.0546 (5)	
O2	0.51059 (16)	0.2973 (2)	0.58981 (11)	0.0384 (4)	
O3	0.4263 (2)	0.0404 (3)	0.90667 (14)	0.0489 (4)	
O4	0.0735 (3)	−0.0003 (3)	0.84929 (18)	0.0662 (6)	
H4	0.164 (5)	−0.035 (7)	0.855 (4)	0.093 (15)*	
C1	0.0892 (3)	0.1905 (4)	0.8793 (2)	0.0509 (6)	
H1	−0.0187	0.2346	0.8966	0.061*	
C2	0.2005 (3)	0.2157 (5)	0.98982 (19)	0.0541 (6)	
H2A	0.179	0.3351	1.024	0.065*	
H2B	0.176	0.1214	1.0462	0.065*	
C3	0.3763 (3)	0.2043 (4)	0.96754 (17)	0.0445 (5)	
H3	0.4521	0.2468	1.0319	0.053*	
C4	0.4356 (2)	0.2197 (3)	0.84932 (16)	0.0356 (4)	
C5	0.3118 (2)	0.2328 (3)	0.74257 (15)	0.0303 (4)	
H5	0.2925	0.1061	0.7149	0.036*	
C6	0.3554 (2)	0.3442 (3)	0.63683 (15)	0.0316 (4)	
H6	0.3543	0.4761	0.6561	0.038*	
C7	0.2322 (2)	0.3066 (3)	0.53335 (16)	0.0364 (4)	
H7	0.2211	0.1723	0.5256	0.044*	
C8	0.0672 (3)	0.3830 (4)	0.55853 (19)	0.0475 (6)	
H8A	−0.0119	0.3553	0.4939	0.057*	
H8B	0.0733	0.516	0.5679	0.057*	
C9	0.0161 (2)	0.2934 (4)	0.67131 (19)	0.0490 (6)	
H9A	−0.0068	0.1639	0.6561	0.059*	
H9B	−0.0837	0.351	0.6926	0.059*	
C10	0.1444 (2)	0.3084 (3)	0.77649 (17)	0.0385 (5)	
C11	0.3227 (3)	0.3750 (3)	0.43019 (17)	0.0395 (5)	
H11	0.3129	0.5097	0.4256	0.047*	
C12	0.4968 (3)	0.3260 (3)	0.47164 (16)	0.0382 (4)	
C13	0.2728 (4)	0.2936 (5)	0.31047 (19)	0.0603 (7)	
H13A	0.3448	0.3376	0.2544	0.09*	
H13B	0.1635	0.3305	0.2868	0.09*	
H13C	0.2785	0.1613	0.3146	0.09*	
C14	0.1588 (3)	0.5107 (4)	0.8180 (2)	0.0560 (6)	
H14A	0.0679	0.5408	0.8623	0.084*	
H14B	0.1595	0.5908	0.7512	0.084*	
H14C	0.2578	0.5264	0.8665	0.084*	
C15	0.6070 (3)	0.2836 (5)	0.83987 (19)	0.0543 (7)	
H15A	0.6664	0.273	0.915	0.081*	
H15B	0.6066	0.4102	0.8148	0.081*	
H15C	0.6578	0.2086	0.7838	0.081*	

### Atomic displacement parameters (Å^2^)

**Table:**

	*U*^11^	*U*^22^	*U*^33^	*U*^12^	*U*^13^	*U*^23^
O1	0.0602 (10)	0.0587 (10)	0.0481 (8)	0.0041 (9)	0.0264 (7)	0.0041 (9)
O2	0.0331 (6)	0.0470 (8)	0.0366 (7)	0.0030 (7)	0.0118 (5)	0.0026 (7)
O3	0.0554 (9)	0.0485 (10)	0.0431 (8)	0.0042 (8)	0.0058 (6)	0.0103 (7)
O4	0.0588 (12)	0.0673 (13)	0.0729 (13)	−0.0278 (11)	0.0077 (9)	0.0140 (11)
C1	0.0335 (10)	0.0708 (18)	0.0504 (12)	−0.0031 (12)	0.0166 (9)	0.0062 (12)
C2	0.0484 (12)	0.0761 (18)	0.0400 (10)	−0.0032 (14)	0.0186 (9)	0.0033 (12)
C3	0.0437 (11)	0.0569 (15)	0.0332 (9)	−0.0071 (11)	0.0051 (8)	0.0002 (10)
C4	0.0323 (9)	0.0401 (11)	0.0350 (9)	0.0002 (9)	0.0069 (7)	0.0001 (9)
C5	0.0261 (8)	0.0312 (9)	0.0342 (9)	−0.0001 (8)	0.0076 (6)	−0.0023 (8)
C6	0.0293 (8)	0.0328 (10)	0.0336 (9)	0.0012 (8)	0.0080 (7)	−0.0007 (7)
C7	0.0381 (10)	0.0341 (10)	0.0374 (9)	0.0014 (9)	0.0042 (7)	0.0025 (9)
C8	0.0364 (10)	0.0569 (15)	0.0489 (11)	0.0073 (10)	−0.0006 (8)	0.0046 (11)
C9	0.0271 (8)	0.0639 (15)	0.0565 (12)	0.0041 (10)	0.0061 (8)	0.0066 (12)
C10	0.0286 (8)	0.0460 (12)	0.0422 (9)	0.0023 (9)	0.0112 (7)	0.0010 (10)
C11	0.0495 (11)	0.0351 (10)	0.0341 (9)	−0.0003 (9)	0.0052 (8)	0.0014 (8)
C12	0.0457 (10)	0.0329 (10)	0.0374 (10)	−0.0004 (9)	0.0127 (8)	0.0014 (8)
C13	0.0753 (16)	0.0665 (17)	0.0382 (11)	0.0005 (15)	−0.0017 (10)	−0.0045 (13)
C14	0.0557 (14)	0.0527 (15)	0.0622 (15)	0.0166 (12)	0.0215 (11)	−0.0093 (12)
C15	0.0320 (9)	0.089 (2)	0.0418 (10)	−0.0093 (12)	0.0040 (8)	0.0017 (13)

### Geometric parameters (Å, °)

**Table:**

O1—C12	1.202 (2)	C7—C8	1.516 (3)
O2—C12	1.364 (2)	C7—C11	1.524 (3)
O2—C6	1.464 (2)	C7—H7	0.98
O3—C3	1.451 (3)	C8—C9	1.530 (3)
O3—C4	1.459 (3)	C8—H8A	0.97
O4—C1	1.427 (4)	C8—H8B	0.97
O4—H4	0.78 (4)	C9—C10	1.547 (3)
C1—C2	1.517 (3)	C9—H9A	0.97
C1—C10	1.548 (3)	C9—H9B	0.97
C1—H1	0.98	C10—C14	1.541 (4)
C2—C3	1.493 (3)	C11—C13	1.519 (3)
C2—H2A	0.97	C11—C12	1.521 (3)
C2—H2B	0.97	C11—H11	0.98
C3—C4	1.475 (3)	C13—H13A	0.96
C3—H3	0.98	C13—H13B	0.96
C4—C15	1.500 (3)	C13—H13C	0.96
C4—C5	1.536 (3)	C14—H14A	0.96
C5—C6	1.518 (2)	C14—H14B	0.96
C5—C10	1.561 (2)	C14—H14C	0.96
C5—H5	0.98	C15—H15A	0.96
C6—C7	1.527 (3)	C15—H15B	0.96
C6—H6	0.98	C15—H15C	0.96
			
C12—O2—C6	108.47 (14)	C7—C8—C9	108.19 (19)
C3—O3—C4	60.93 (13)	C7—C8—H8A	110.1
C1—O4—H4	103 (4)	C9—C8—H8A	110.1
O4—C1—C2	110.8 (2)	C7—C8—H8B	110.1
O4—C1—C10	112.2 (2)	C9—C8—H8B	110.1
C2—C1—C10	111.9 (2)	H8A—C8—H8B	108.4
O4—C1—H1	107.2	C8—C9—C10	114.25 (18)
C2—C1—H1	107.2	C8—C9—H9A	108.7
C10—C1—H1	107.2	C10—C9—H9A	108.7
C3—C2—C1	112.77 (17)	C8—C9—H9B	108.7
C3—C2—H2A	109	C10—C9—H9B	108.7
C1—C2—H2A	109	H9A—C9—H9B	107.6
C3—C2—H2B	109	C14—C10—C9	109.8 (2)
C1—C2—H2B	109	C14—C10—C1	108.05 (19)
H2A—C2—H2B	107.8	C9—C10—C1	109.22 (19)
O3—C3—C4	59.79 (13)	C14—C10—C5	111.13 (18)
O3—C3—C2	116.2 (2)	C9—C10—C5	110.48 (16)
C4—C3—C2	122.93 (18)	C1—C10—C5	108.08 (18)
O3—C3—H3	115.4	C13—C11—C12	112.21 (19)
C4—C3—H3	115.4	C13—C11—C7	117.1 (2)
C2—C3—H3	115.4	C12—C11—C7	100.87 (15)
O3—C4—C3	59.28 (14)	C13—C11—H11	108.7
O3—C4—C15	112.81 (18)	C12—C11—H11	108.7
C3—C4—C15	117.85 (17)	C7—C11—H11	108.7
O3—C4—C5	111.03 (17)	O1—C12—O2	120.52 (19)
C3—C4—C5	119.16 (16)	O1—C12—C11	128.83 (18)
C15—C4—C5	119.95 (17)	O2—C12—C11	110.63 (15)
C6—C5—C4	118.90 (15)	C11—C13—H13A	109.5
C6—C5—C10	106.09 (15)	C11—C13—H13B	109.5
C4—C5—C10	111.90 (15)	H13A—C13—H13B	109.5
C6—C5—H5	106.4	C11—C13—H13C	109.5
C4—C5—H5	106.4	H13A—C13—H13C	109.5
C10—C5—H5	106.4	H13B—C13—H13C	109.5
O2—C6—C5	115.68 (15)	C10—C14—H14A	109.5
O2—C6—C7	102.98 (14)	C10—C14—H14B	109.5
C5—C6—C7	109.82 (16)	H14A—C14—H14B	109.5
O2—C6—H6	109.4	C10—C14—H14C	109.5
C5—C6—H6	109.4	H14A—C14—H14C	109.5
C7—C6—H6	109.4	H14B—C14—H14C	109.5
C8—C7—C11	121.77 (19)	C4—C15—H15A	109.5
C8—C7—C6	110.13 (17)	C4—C15—H15B	109.5
C11—C7—C6	101.85 (16)	H15A—C15—H15B	109.5
C8—C7—H7	107.4	C4—C15—H15C	109.5
C11—C7—H7	107.4	H15A—C15—H15C	109.5
C6—C7—H7	107.4	H15B—C15—H15C	109.5
			
O4—C1—C2—C3	79.3 (3)	C11—C7—C8—C9	−176.7 (2)
C10—C1—C2—C3	−46.7 (3)	C6—C7—C8—C9	−57.7 (3)
C4—O3—C3—C2	114.5 (2)	C7—C8—C9—C10	52.5 (3)
C1—C2—C3—O3	−53.5 (3)	C8—C9—C10—C14	69.4 (3)
C1—C2—C3—C4	16.1 (4)	C8—C9—C10—C1	−172.3 (2)
C3—O3—C4—C15	109.9 (2)	C8—C9—C10—C5	−53.5 (3)
C3—O3—C4—C5	−112.26 (18)	O4—C1—C10—C14	−179.6 (2)
C2—C3—C4—O3	−103.4 (3)	C2—C1—C10—C14	−54.3 (3)
O3—C3—C4—C15	−101.3 (2)	O4—C1—C10—C9	61.0 (3)
C2—C3—C4—C15	155.3 (3)	C2—C1—C10—C9	−173.8 (2)
O3—C3—C4—C5	98.4 (2)	O4—C1—C10—C5	−59.3 (2)
C2—C3—C4—C5	−4.9 (4)	C2—C1—C10—C5	66.0 (3)
O3—C4—C5—C6	−146.38 (16)	C6—C5—C10—C14	−65.2 (2)
C3—C4—C5—C6	148.0 (2)	C4—C5—C10—C14	66.0 (2)
C15—C4—C5—C6	−11.9 (3)	C6—C5—C10—C9	57.0 (2)
O3—C4—C5—C10	89.3 (2)	C4—C5—C10—C9	−171.85 (19)
C3—C4—C5—C10	23.7 (3)	C6—C5—C10—C1	176.44 (18)
C15—C4—C5—C10	−136.2 (2)	C4—C5—C10—C1	−52.4 (2)
C12—O2—C6—C5	148.07 (17)	C8—C7—C11—C13	−81.2 (3)
C12—O2—C6—C7	28.3 (2)	C6—C7—C11—C13	155.8 (2)
C4—C5—C6—O2	52.5 (2)	C8—C7—C11—C12	156.7 (2)
C10—C5—C6—O2	179.57 (16)	C6—C7—C11—C12	33.8 (2)
C4—C5—C6—C7	168.51 (17)	C6—O2—C12—O1	175.0 (2)
C10—C5—C6—C7	−64.4 (2)	C6—O2—C12—C11	−6.3 (2)
O2—C6—C7—C8	−169.05 (17)	C13—C11—C12—O1	34.9 (4)
C5—C6—C7—C8	67.2 (2)	C7—C11—C12—O1	160.3 (3)
O2—C6—C7—C11	−38.5 (2)	C13—C11—C12—O2	−143.7 (2)
C5—C6—C7—C11	−162.29 (16)	C7—C11—C12—O2	−18.2 (2)

### Hydrogen-bond geometry (Å, °)

**Table:**

*D*—H···*A*	*D*—H	H···*A*	*D*···*A*	*D*—H···*A*
O4—H4···O3	0.79 (4)	2.27 (4)	2.952 (3)	146 (5)
C9—H9A···O4	0.97	2.55	2.958 (3)	105
C5—H5···O4	0.98	2.57	2.925 (3)	101
C15—H15C···O2	0.96	2.53	2.913 (3)	104
